# The Expression Levels of CD20 as a Prognostic Value in Feline B-Cell Nasal Lymphoma: A Pilot Study

**DOI:** 10.3390/ani14071043

**Published:** 2024-03-29

**Authors:** Kravee Chaipoca, Theerapol Sirinarumitr, Supreeya Srisampan, Charuwan Wongsali, Attawit Kovitvadhi, Tassanee Jaroensong

**Affiliations:** 1 Department of Companion Animal Clinical Sciences, Faculty of Veterinary Medicine, Kasetsart University, 50 Ngamwongwan Rd., Lat Yao, Chatuchak, Bangkok 10900, Thailand; kravee.c@ku.th; 2 Department of Pathology, Faculty of Veterinary Medicine, Kasetsart University, 50 Ngamwongwan Rd., Lat Yao, Chatuchak, Bangkok 10900, Thailand; fvettps@yahoo.com; 3 Center for Veterinary Diagnostic Laboratory-Bangkhen, Faculty of Veterinary Medicine, Kasetsart University, 50 Ngamwongwan Rd., Lat Yao, Chatuchak, Bangkok 10900, Thailand; yyungvet@gmail.com (S.S.); fvetcrw@ku.ac.th (C.W.); 4 Department of Physiology, Faculty of Veterinary Medicine, Kasetsart University, 50 Ngamwongwan Rd., Lat Yao, Chatuchak, Bangkok 10900, Thailand; fvetawk@ku.ac.th; 5 Feline Unit, Kasetsart University Veterinary Teaching Hospital, Faculty of Veterinary Medicine, Kasetsart University, 50 Ngamwongwan Rd., Lat Yao, Chatuchak, Bangkok 10900, Thailand

**Keywords:** cats, CD20, immunophenotype, nasal lymphoma

## Abstract

**Simple Summary:**

Most cats with nasal lymphoma have a B-cell phenotype with CD20 expression. The common treatment for feline nasal lymphoma involves radiotherapy and cyclophosphamide, vincristine, and prednisolone (COP) chemotherapy. This retrospective study investigates 27 cats with nasal lymphoma. After the second and sixth weeks of treatment, the cats showed a reduction in red blood cell count. This study revealed that cats with tumors confined to a single nasal passage and exhibiting high CD20 expression had significantly longer survival times compared to other groups.

**Abstract:**

The effect of the semi-quantitative expression of CD20 in the prognosis of feline nasal lymphoma has not been described. This study investigated the prognostic significance of CD20 expression, clinicopathological characterization, and treatment outcomes in cats with nasal lymphoma. Clinical data from cats diagnosed with nasal lymphoma were retrospectively collected, including signalment, clinical signs, clinicopathological variables, treatment outcomes, and survival times. Using ImageJ software, CD20 expression was semi-quantitatively measured based on the proportion of CD20-positive areas. Correlations between laboratory findings, immunohistochemical expressions, and survival outcomes were investigated. All cats included in the study exhibited the B-cell immunophenotype. During treatment, a reduction in PCV was noted in the cats at the second and sixth weeks (*p* = 0.01 and *p* = 0.01, respectively). The cats with low CD20 expression exhibited a significantly shorter MST (91 days; 95% CI, 41–141) than those with high CD20 expression (MST, 214 days; 95% CI, 76–351) (*p* = 0.01). Stage T1 cats displayed a higher MST (143 days; 95% CI, 144–172) than those in other stages > T1 (120 days, 95% CI, 71–169 days) (*p* = 0.04). Anemia, a common adverse effect in feline nasal lymphoma, did not impact MST. T1 clinical staging and high CD20 expression showed a trend for better MST.

## 1. Introduction

Feline lymphoma accounts for approximately 30% of tumors in cats [1,2], and feline nasal lymphoma represents about 63% of all extranodal lymphomas in these animals [3,4,5]. Clinical signs of nasal lymphoma include chronic sneezing, mucoid to purulent or bloody nasal discharge, snoring or dyspnea due to nasal obstruction, epiphora, exophthalmos, facial deformities, anorexia, and weight loss, which are similar to the symptoms of other respiratory diseases [6,7,8,9]. Feline lymphoma commonly occurs in older, feline leukemia virus (FeLV)-negative cats [10]. Treatment options for this type of lymphoma include chemotherapy, radiotherapy, and combined chemotherapy and radiotherapy [11,12,13]. The median survival time (MST) for cats with nasal lymphoma ranged widely post-treatment. Cats suffering from nasal lymphoma treated with radiotherapy are more likely to have a higher MST than those treated with chemotherapy alone [9]. However, there was no difference found in progression-free survival among chemotherapy, radiotherapy, and combined chemotherapy and radiotherapy [7]. Immunophenotyping of feline nasal lymphoma, using antibody markers for T and B lymphocytes to stain paraffin tissue sections, has exhibited primarily the B-cell phenotype, followed by T-cell lymphoma and a mixed population of B-cells and T-cells [14,15]. In contrast to cats, nasal lymphoma is uncommon in humans, with the majority being of T-cell origin, while primary nasal B-cell lymphoma is uncommon [16,17,18].

Although previous studies have shown that the clinicopathological data, histopathology, and immunophenotype of feline lymphoma predominantly involve the B-cell phenotype [5,14,15], identifying the immunophenotype remains essential for further prognostic evaluation and treatment options. Researchers in immunology have focused on developing monoclonal anti-CD 20 antibodies for immunotherapies targeting B-cell lymphoma, as the CD20 antigen is broadly expressed in both B-cell lymphoma and normal mature B-cells [19,20,21,22]. The monoclonal anti-CD20 antibody has been approved in human oncology medicine to treat patients with B-cell lymphoma, as this antibody is believed to recruit immune system cells to attack B-cells, resulting in the depletion of B-cells for several months [23]. Adding monoclonal anti-CD20 antibodies to multiple-drug chemotherapy regimens for treating B-cell lymphoma significantly improves outcomes. Thus, understanding the relevance of immunophenotype may be crucial in considering immunotherapeutic options and prognosis.

The objective of this retrospective study was to investigate the prognostic significance of CD20 expression, clinicopathological characterization, and treatment outcomes in cats with nasal lymphoma.

## 2. Materials and Methods

### 2.1. Case Selection

This study investigated client-owned cats with nasal lymphoma from the archives of the Kasetsart University Veterinary Teaching Hospital, Bangkok, Thailand, between August 2017 and August 2022. The cats were included if they had a complete medical record and histological diagnosis of nasal lymphoma. Data collection from the medical records included the presented complaint, physical examination findings, anatomical distribution, clinicopathological results, histopathological results, adverse events of treatment (modified according to the Veterinary Cooperative Oncology Group-Common Terminology Criteria for Adverse Events (VCOG-CTCAE v2) [24]), and survival time. The exclusion criterion was defined as a lack of contact with the owner after sample collection. The classification of cats with lymphoma was based on the anatomical distribution of the primary tumor lesions. Cats were staged in accordance with the modified Adam’s staging system for canine nasal tumors [12,25], categorized as follows: stage T1, confined to one nasal passage; stage T2, any bony involvement (beyond turbinate), but with no evidence of orbital/subcutaneous/submucosal mass; stage T3, orbital involvement or nasopharyngeal or subcutaneous or submucosal mass; and stage T4, tumor causing lysis of the cribriform plate [26]. Radiological examination using a CT Scanner (GE Optima CT660, GE Healthcare, Milwaukee, WI, USA) and ultrasonography using a real-time scanner (LOGIQ E9, GE, Fairfield, CT, USA) were performed in all cases to assess thoracic and abdominal metastasis, respectively.

### 2.2. Clinicopathologic Evaluations

Complete blood count and biochemical values were compared during the pre-treatment phase, as well as between the second and sixth weeks of the treatment period. The evaluated hematological parameters were packed cell volume (PCV), white blood cell count (WBC), neutrophil count, monocyte count, and lymphocyte count, determined using a Sysmex XN-1000TM Hematology Analyzer (Sysmex, Mundelein, IL, USA). Serum biochemistry parameters were blood urea nitrogen (BUN), creatinine (Creat), alanine aminotransferase (ALT), total protein (TP), and albumin (ALB), determined using an IL Lab 650 chemistry system (Diamond Diagnostics, Holliston, MA, USA). Screening for retroviral infection (FeLV antigen and FIV antibody) was performed using a rapid immune migration-based (WITNESS ^®^ FeLV-FIV) point-of-care test kit.

### 2.3. Histopathology and Immunohistochemistry

All tumor tissues were obtained through incisional biopsy, then fixed in 10% neutral buffer formalin and embedded in paraffin. Sections (3 μm) were stained using hematoxylin and eosin (H&E). The H&E-stained sections were evaluated by Thai Board pathologists to diagnose and characterize the morphological descriptions. For histopathology and immunohistochemistry (IHC) staining after deparaffinization and rehydration, antigen retrieval was achieved via immersion in a citrate-based buffer containing surfactant (pH~6.0) for 45 min in a vegetable steamer. Hydrogen peroxide (3% *v*/*v*) was applied to tissue sections for 5 min at room temperature to inactivate endogenous peroxidase. The slides were incubated at 37 °C for 60 min with mouse monoclonal anti-human-CD3 (dilution 1 in 100; Leica Biosystem, Newcastle-upon-Tyne, UK) [27] and rabbit monoclonal anti-human-CD20 (dilution 1 in 200; Thermo Scientific, Rockford, IL, USA) [28] as the primary antibodies. Immunolabelling was performed using Novolink Polymer detection (Leica Biosystems; Newcastle-upon-Tyne, UK). Finally, the reactions were visualized with DAB as a chromogen; sections were counterstained with hematoxylin. A normal lymph node was used as the positive control, whereas the negative control was conducted on slides without the primary antibody. A semi-quantitative analysis of the positive area was conducted on each lymphoma tissue (excluding necrosis, fibrosis, hemorrhage, and blood vessels in the tissue) at 400× magnification on ImageJ software (http:/rsbweb.nih.gov/ij/) (National Institute of Health; Bethesda, MD, USA) [29,30,31,32]. Then, the proportion of CD20-positive labelling in the membrane among the total of neoplastic lymphoid cells was calculated. The IHC staining in each tissue was scored as follows: (−) defined as <25% of the positive area; (+) defined as between 25% and <50%; (++) defined as between 50% and <75%; (+++) defined as between 75% and ≤90%; and (++++) defined as >90% [32].

### 2.4. Treatment

The cats included in this study were treated using various treatment modalities, consisting of cyclophosphamide, vincristine, and prednisolone (COP) chemotherapy or radiotherapy. Detailed treatment protocols were recorded, which included information such as the specific type of COP chemotherapy administered, the dosage, the type of radiotherapy equipment utilized, the total radiation dose delivered, and the duration of the treatment. COP chemotherapy was used for cats within the induction phase during weeks 1, 2, 3, and 4 with vincristine 0.5–0.6 mg/m^2^ administered intravenously once a week, combined with cyclophosphamide 250 mg/m^2^ administered orally in weeks 1 and 4. Then, a maintenance phase followed which involved administering vincristine and cyclophosphamide every 3 weeks, continuing until week 52 or 1 year. Prednisolone was used on the first day at a dosage of 2 mg/kg orally once a day. Then, prednisolone was continued and its dosage gradually tapered off over 1 year [33,34,35].

For radiotherapy, cats received radiotherapy with the hypofractionated protocol using a megavoltage radiation machine. Radiotherapy treatment planning was carried out using a CT Scanner (GE Optima CT660, GE Healthcare, WI, USA). Radiotherapy on cats with nasal lymphoma was scheduled weekly and administered using a 4 MV X-ray linear accelerator (The Varian Clinac 2100C, Varian Medical System Inc., Palo Alto, CA, USA). Throughout all of the processes, cats were anesthetized and positioned prone. Initial treatment lasted for a duration of 8 weeks. Then, cats underwent monitoring for treatment response in the third and sixth months after the last radiotherapy session.

### 2.5. Statistical Analysis

The MST for cats diagnosed with lymphoma was calculated using a Kaplan–Meier survival curve. Hematology and blood chemistry were analyzed using one-way repeated-measures ANOVA, with the Bonferroni test used as a post hoc analysis, with time as the within-subjects factor. Cox proportional hazard regression analysis was employed to assess associations with prolonged survival, considering various factors such as the stage of the tumors, treatment options, PCV, and CD20 expression. The significance level for all tests was set at *p* ≤ 0.05. All statistical analyses were performed using R statistics within RStudio Version 2023.06.2+561. Survival analysis was conducted using the ‘survival’ package, while one-way repeated-measures ANOVA was executed using the ‘Rcmdr’ package. Data were visualized using the ‘ggplot2’ package. The censor was defined as an instance where the animal was still alive but had either disappeared or data collection ceased, meaning complete information was unavailable.

## 3. Results

Thirty-seven cats with histologically diagnosed nasal lymphoma were identified, and twenty-seven cats met the inclusion criteria. Ten cats were excluded from the analysis due to a loss of contact with the owner following sample collection. The characteristics of the 27 cats are presented in Table 1. Of the 27 cats, 19 cats were male, and 8 cats were female. The mean age of the cats diagnosed with nasal lymphoma was 10 years, with a range of 3 to 16 years. The mean body weight of these cats was 5.49 kg, with a range of 2.15 to 7.30 kg. The study comprised predominantly domestic shorthair cats (*n* = 22, 81.48%). Other breeds included three Maine Coons, one Scottish Fold, and one Persian. FeLV and FIV serology results were obtained. Among the cats, four (14.81%) tested positive for FeLV, and three (11.11%) tested positive for FIV. Additionally, 1 cat (3.70%) was positive for both FeLV and FIV, while the remaining 19 cats (70.37%) tested negative for both infections. The major presenting symptoms for the cats were nasal discharge (*n* = 19, 70.37%), nasal swelling (*n* = 10, 37.04%), dyspnea (*n* = 9, 33.33%), partial airflow (*n* = 6, 22.22%), stridor (*n* = 6, 22.22%), ocular discharge (*n* = 4, 14.81%), sneezing (*n* = 3, 11.11%), exophthalmos (*n* = 2, 7.41%), third eyelid prolapse (*n* = 2, 7.41%), conjunctival swelling (*n* = 1, 3.70%), submandibular lymph node enlargement (*n* = 1, 3.70%), and swelling of the cranial hard palate (*n* = 1, 3.70%). According to the modified Adam’s staging system for canine nasal tumors, the cats were classified as stages T1 (*n* = 12, 44.44%), T2 (*n* = 4, 14.81%), T3 (*n* = 9, 33.33%), and T4 (*n* = 1, 3.70%). There was one (3.70%) cat whose tumor was not staged.

**Figure 1:**
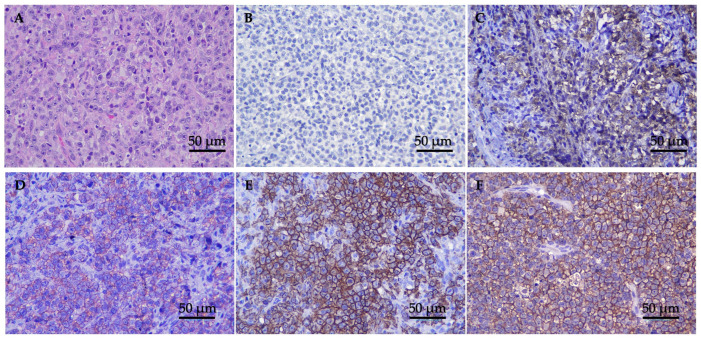
H&E and IHC staining of nasal lymphoma tissue; (**A**) tumor tissue stained with H&E 400× magnification; (**B**–**F**) classification of immunophenotype expression by IHC (original 400× magnification). Panels show examples of cases categorized in each score: (**B**) anti-CD3 negative expression (−) = expression level < 25% of the positive area; (**C**) anti-CD20 low positive (+) = expression level between 25% and <50%; (**D**) anti-CD20 slightly positive (++) = expression level between 50% and <75%; (**E**) anti-CD20 moderately positive (+++) = expression between 75% and ≤90; and (**F**) anti-CD20 highly positive (++++) = expression level > 90%.

Twenty-seven cats with histopathological analysis revealed a microscopic appearance with densely packed, unencapsulated, and poorly demarcated nasal mucosa tissue, invading submucosa, and highly cellular neoplasm. The population predominantly comprised atypical discrete large round cells with generally distinct cell borders and moderate eosinophilic cytoplasm. Tumor cells were round vesicular nuclear with nuclear indentation. The nuclei were round, coarse chromatin with enlarged nucleoli. There were mitoses of approximately 10–15 cells/high-power fields and atypical mitosis was commonly found (Figure 1A).

Immunohistochemistry was performed on all 27 tumors, and the results are summarized in Table 2. Based on the results, all tumors showed a negative T-cell marker and a positive B-cell marker. Eighteen cats (66.67%) showed low CD20 expression ((+), *n* = 5, 18.52%; (++), *n* = 13, 48.15%), while nine cats (33.33%) showed high CD20 expression ((+++), *n* = 8, 29.63%; (++++), *n* = 1, 3.70%) (Figure 1B–F). The median values of the positive ratios of the CD20 expression levels were as follows: low CD20 expression was 40.15% (range, 26.44–73.42%), while high CD20 expression was 83.44% (range, 75.68–90.74%). In the cats with low CD20 expression, the tumor staging was as follows: nine cats (50.00%) were classified as stage T1, two cats (11.011%) as stage T2, six cats (33.33%) as stage T3, and one cat (5.56%) was not staged. Meanwhile, among the cats with high CD20 expression, the tumor staging was as follows: three cats (33.33%) were classified as stage T1, two cats (22.22%) as stage T2, three cats (33.33%) as stage T3, and one cat (11.11%) as stage T4.

**Figure 2:**
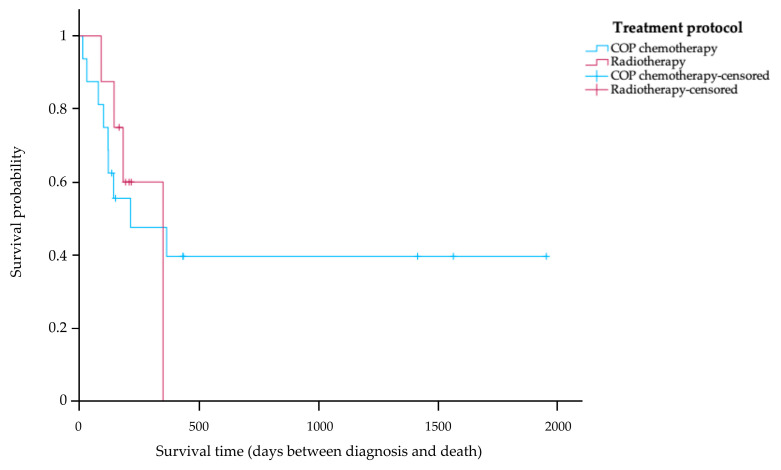
Kaplan–Meier survival curve showing survival time of treated cats with nasal lymphoma stratified according to treatment protocol. The blue line indicates cats that received a COP chemotherapy; the red line indicates cats that received radiotherapy. There was no significant difference in survival time between the two groups (*p* = 0.50).

Of the 27 cats in the study, 24 cats (88.89%) received treatment after their diagnosis. Of the cats in the treatment group, 5 cats (20.83%) are still alive, 13 cats (54.17%) died during treatment, and 6 cats (25.00%) have an unknown survival time. Among the cats receiving treatment, the 16 cats (66.67%) with COP chemotherapy were divided into the following tumor stages: eight cats (50.00%) were classified as stage T1, three cats (18.75%) as stage T2, and five cats (31.25%) as stage T3. The other eight cats (33.33%) that received radiotherapy were divided into the following tumor stages: four cats (50.00%) were classified as stage T1, one cat (12.50%) as T2, and three cats (37.50%) as stage T3. The Kaplan–Meier survival curve in Figure 2 shows that the MST of the 16 cats receiving COP chemotherapy was 214 days (range 14–1955 days). Eight cats were treated with radiotherapy, and their MST was 350 days (range 91–350 days). No statistically significant difference in MST was observed between cats receiving COP chemotherapy and those treated with radiotherapy (*p* = 0.50).

**Figure 3:**
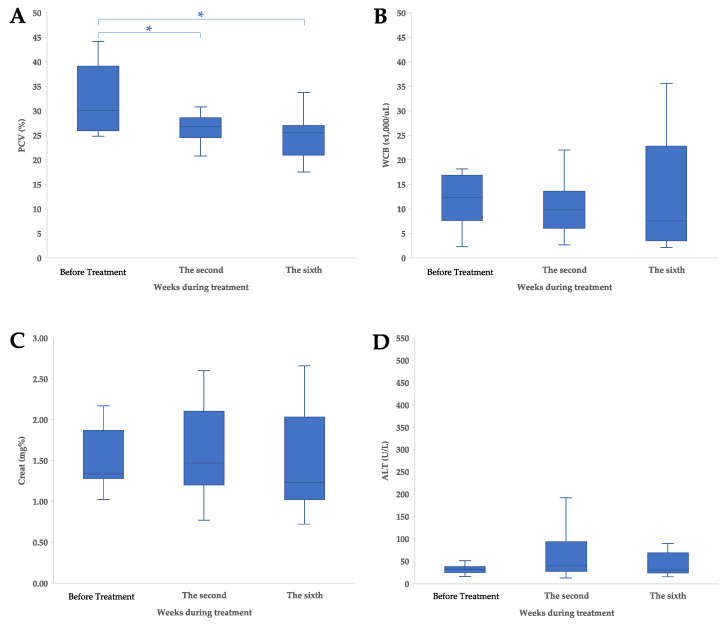
Clinicopathological characteristics and adverse events of treatment in cats with nasal lymphoma; (**A**) pack cell volume (PCV), (**B**) white blood cell, (**C**) creatinine (Creat), and (**D**) alanine aminotransferase (ALT) concentration. The values are shown as data plot and mean (* *p* < 0.05).

The clinicopathological information for the study was obtained from medical records, as shown in Supplementary Table S1. Following the collection of hematological parameter data, the median PCVs for cats before treatment and at the second week and sixth week were 30.1% (range 24.80–44.10%), 26.8% (range 12.30–30.80%), and 25.5% (range 17.60–33.80%), respectively. There was a significant difference in the reduction in PCV after treatment (Figure 3A), as observed at the second (*p* = 0.01) and sixth (*p* = 0.01) weeks.

The median WBCs for cats before treatment and at the second and sixth weeks were 12.36 × 10^3^ cell/μL (range 2.29–18.21 × 10^3^ cell/μL), 9.80 × 10^3^ cell/μL (range 2.69–22.07 × 10^3^ cell/μL), and 7.59 × 10^3^ cell/μL (range 2.10–35.63 × 10^3^ cell/μL), respectively. There was no significant difference in the WBC after treatment (Figure 3B), as observed at the second (*p* = 0.46) and sixth (*p* = 1.00) weeks.

The median Creat levels for cats before treatment, at the second week, and at the sixth week were 1.34 mg% (range 1.02–2.17 mg%), 1.47 mg% (range 0.77–2.60 mg%), and 1.23 mg% (range 0.72–2.66 mg%), respectively. There was no significant difference in the Creat level after treatment (Figure 3C), as observed at the second (*p* = 1.00) and sixth (*p* = 1.00) weeks.

The median ALTs for cats before treatment, at the second week, and at the sixth week were 32 IU/L (range 17–512 IU/L), 40 IU/L (range 13–192 IU/L), and 31 IU/L (range 16–90 IU/L), respectively. There was no significant difference in the ALT after treatment (Figure 3D), as observed at the second (*p* = 1.00) and sixth (*p* = 1.00) weeks.

**Figure 4:**
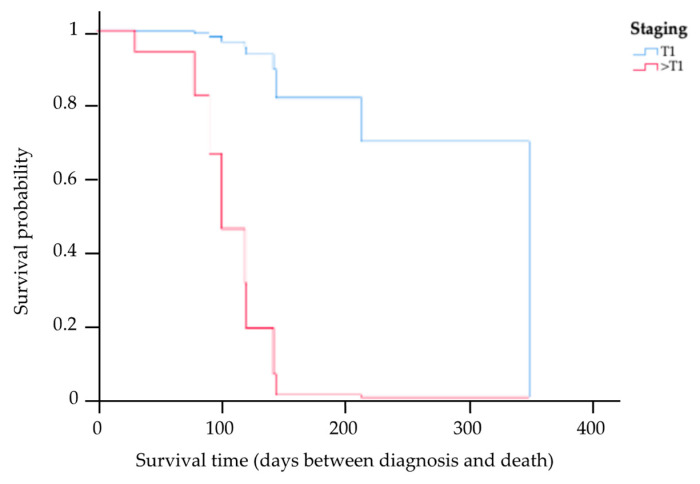
Kaplan–Meier survival curve showing the MST for cats with nasal lymphoma in stage T1 and other staging categories, where the blue line indicates cats with stage T1, and the red line indicates cats with the other staging categories (>T1). There was a significant difference in the MST between the two groups (*p* = 0.04).

**Figure 5:**
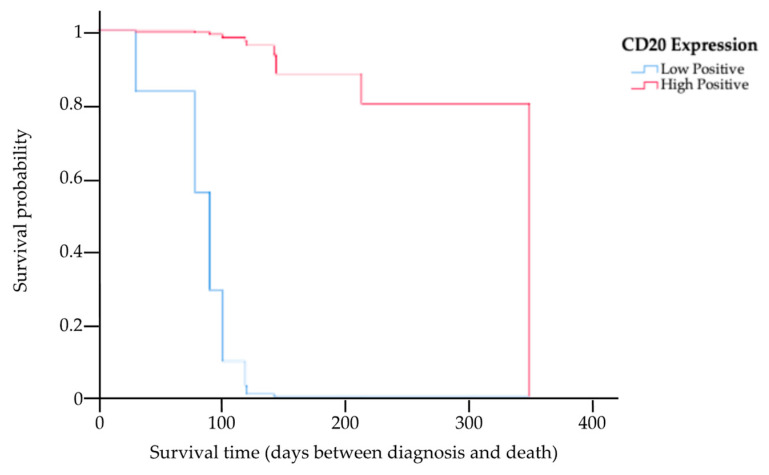
Kaplan–Meier survival curve showing the MST for cats with low and high expression of CD20, where the blue line indicates cats with low CD20 expression, and the red line indicates cats with high CD20 expression. There was a significant difference in the MST between the two groups (*p* = 0.01).

The results of the multivariate analysis of prognostic factors based on the Cox proportional hazards model of the 13 cats for whom complete information on staging, treatment options, PCV, CD20 expression level, and survival times were available are provided in Table 3. This study showed the MST had a trend of being higher among cats in stage T1 compared to those in other staging categories (HR 0.04, *p* = 0.04). Kaplan–Meier survival curves showing the MST for cats with nasal lymphoma in stage T1 and in other staging categories are presented in Figure 4. There was a significant difference in the MST between the two groups (*p* = 0.04). Based on the treatments, four cats received initial local radiation therapy, with a total radiation dose in the range of 36–48 Gy delivered to the tumor. In total, nine cats exclusively underwent systemic treatment, encompassing the administration of COP chemotherapy, resulting in an MST of 121 days (range 31–350). The MST for cats that underwent radiotherapy was 145 days (range 91–208 days). However, there were no significant differences in the MST between the COP chemotherapy and radiotherapy groups (*p* = 0.18). In addition, an analysis was also conducted to determine differences in the MSTs of cats with anemia (PCV ≤ 25%) and cats without anemia (PCV > 25%). This study found that there was no significant difference in the MST between these two groups (*p* = 0.96). However, the MST in cats with low CD20 expression was significantly shorter (121-fold) than in cats with high CD20 expression (HR 120.93, *p* = 0.01). The Kaplan–Meier survival curves showing the survival times for cats with low and high CD20 expression are presented in Figure 5.

## 4. Discussion

This study described the nature and immunohistochemical expression of feline nasal lymphoma, as well as the efficacy of palliative radiotherapy and COP chemotherapy and adverse effects of treatment, to better determine its prognosis. The median age of the cats in the study was 10 years, which was similar to the findings of earlier investigations in the UK and the USA [12,15,36]. There was a higher male-to-female ratio (2:1). The results of the current study are similar to other studies [2,14,37,38]. Although there was only a small number of individuals in our study, making meaningful statistical analysis difficult, domestic shorthair cat breeds were over-represented. This outcome was not surprising, given that this study encompassed as much as 81.48% of the cat population under investigation, reflecting the high population of this breed in Thailand. The most predominant clinical manifestations observed were nasal discharge, stridor, partial airflow, and nasal swelling. These symptoms are common in nasal tumors [5,7,12,14,15,36].

Of particular note, the cats with nasal lymphoma in this study always had a B-cell phenotype that was negative for retroviral infections (70.37%). It is possible that FeLV proviral insertion in a proportion of feline lymphoma tissues is more common in lymphomas of T-cell origin [10,39].

In addition, considering the immunophenotype of the lymphomas in this study, it was notable that B-cell lymphomas were more pronounced than the other immunophenotypes, aligning with findings from previous studies [13,25,40]. On the other hand, other studies reported that T-cell phenotypes were more prominent than B-cell phenotypes [5,41]. In human medicine, there is a notable predominance of T-cell nasal lymphomas (75%), whereas B-cell nasal lymphomas present at a rate of 25% [17]. Our IHC results showed a high expression of CD20 antigens in feline nasal lymphoma tissues. The amount of CD20 expression in this study was measured using the ImageJ software and had not previously been studied. CD20 expression is considered a reliable marker for B-cells in IHC diagnosis of human and feline lymphoma [16,17,25,40,42,43], and it has been a target for immunotherapy that has been accessible for several years in the treatment of human patients with B-cell non-Hodgkin’s lymphoma.

This study identified no significant differences in the MST between the cats treated with chemotherapy and those subjected to radiotherapy (*p* = 0.50). The results are consistent with previous studies indicating that treatment with chemotherapy or radiotherapy yielded no significant difference in the MST. Notably, cats treated with radiotherapy tended to have a longer survival time compared to those treated with chemotherapy, a trend consistent with prior studies, with an extension in survival of approximately 1 year [7,12]. Although this study did not analyze the stages of nasal lymphoma according to COP chemotherapy or radiotherapy, some studies found that cats without brain involvement receiving radiotherapy have a longer survival time than those with brain involvement [8,12]. It is crucial to acknowledge that this study is subject to limitations, particularly the relatively small number of cats treated with radiotherapy in the retrospective sample.

The adverse effects of both treatments were the relatively high incidence of a reduction in PCV, which was identified as a negative prognostic factor and usually occurs after treatment [8,9,44]. Reductions in PCV are caused by bone marrow infiltration, chronic gastrointestinal bleeding, or insufficient iron intake. However, PVC in cats with chronic illnesses such as nasal lymphoma can occur due to decreased iron absorption and accumulation in macrophages [45,46]. The change in the median WBC in this study also decreased slowly over the second and sixth weeks, which is consistent with previous studies. However, previous studies did not find significant differences in WBC between different chemotherapy treatments [47,48].

Through multivariable analysis, CD20 expression was identified as a significant prognostic factor. Cats with low CD20 expression displayed shorter MSTs than cats with high CD20 expression. In the development of B-lineage cells, the surface molecule antigen CD20 was found in the development of late pro-B-cells until mature B-cell differentiation [23,49]. This finding regarding the surface antigen should be investigated in order to predict tumor cell proliferation and prognostic factors. There could be potential to utilize the expression of CD19 as a prognostic marker. CD19 enhances B-cell antigen receptor signaling, thereby amplifying tumor cell proliferation and promoting cell survival [23,50,51]. Therefore, using CD20 and CD19 may be useful in prognostication. In addition, the prognostic factors were also related to staging, specifically stage T1, as the modified Adam’s staging system exhibited a significant association with an increase in MST. These results align with findings from other studies, which have shown that the MST tends to decrease in cases involving cats with metastatic conditions, such as cribriform plate destruction [8,12]. However, neither anemia following treatment nor the specific treatment types had a significant prognostic impact, which is consistent with the outcomes of other studies based on multivariate analysis [7,48].

The current study was limited by its retrospective nature, as a complete follow-up of clinical signs, imaging, full staging work-up, and necropsy findings were not available for all cats; the relatively small sample size for statistical analysis was also limiting. This study attributed the deaths of cats with undetermined or unspecified causes of death to lymphoma. Consequently, further studies should be conducted with comprehensive hematological records to examine potential candidate prognostic factors and investigate the potential adverse effects of cancer treatment. Other methods for assessing CD20 expression levels may be alternative approaches, including the percentage of positive immunolabeled cells over the total cells in each selected area [52], evaluating the completeness and intensity of membrane staining added for a score [31], or quantifying corrected pixel density [53]. Furthermore, the nature of the nasal cavity biopsy conducted in cats results in very small tissue samples. Insufficient availability of paraffin-embedded tissue samples may hinder the determination of lymphoid lineage through antigen receptor gene rearrangement, the method recommended for the analysis of feline lymphomas [41]. Nonetheless, the utilization of CD20 proved effective in differentiating between lymphoma immunophenotypes [40,42,54,55]. Alternatively, using other types of immunohistochemistry, such as PAX5, could further enhance characterization efforts [56,57,58].

This study was part of a body of work that is expected to be useful in terms of analyzing CD20 expression levels in feline nasal lymphoma using existing data to find relationships between CD20 expression and survival time. As of now, this study has not studied the immunotherapeutic options, but in the future, this information may be useful in studies of novel treatments.

## 5. Conclusions

This study reported that most feline nasal lymphomas were of the B-cell immunophenotype with expression of CD20. There were no significant differences in the MST between COP chemotherapy and radiotherapy in cats. Moreover, the main adverse effect on the hematology of cats receiving COP chemotherapy and radiotherapy was anemia, which occurred at the second and sixth weeks of the treatment period; however, this condition had no significance on the MST. Cox proportional hazard regression analysis revealed that clinical staging (T1) and CD20 expression were significantly associated with MST. Therefore, CD20 overexpression can be a positive prognostic factor in feline nasal lymphoma. Cats with high CD20 expression had a longer MST than cats with low CD20 expression. As this study found no suitable treatment, future studies are needed to investigate the efficacy of other chemotherapy protocols, multifractionated radiotherapy, and B-cell based immunotherapy for feline nasal lymphoma.

## Figures and Tables

**Table 1:** Patient characteristics of 27 cats with nasal lymphoma.

Variable	Category	Number	Percentage
Gender			
	Male	19	70.37%
	Female	8	29.63%
Age (years)			
	1–6	11	40.74%
	7–10	9	33.33%
	>10	7	25.93%
Body Weight (kg)			
	<4	10	37.04%
	4–6	12	44.44%
	>6	5	18.52%
Breed			
	DSH	22	81.48%
	Maine Coon	3	11.11%
	Scottish Fold	1	3.70%
	Persian	1	3.70%
Retrovirus status			
	FeLV antigen-positive	4	14.81%
	FIV antibody-positive	3	11.11%
	FeLV antigen- and FIV antibody-positive	1	3.70%
	FeLV antigen- and FIV antibody-negative	19	70.37%
Presenting symptoms			
	Nasal discharge	19	70.37%
	Nasal swelling	10	37.04%
	Dyspnea	9	33.33%
	Partial airflow	6	22.22%
	Stridor	6	22.22%
	Ocular discharge	4	14.81%
	Sneezing	3	11.11%
	Exophthalmos	2	7.41%
	Third eyelid prolapse	2	7.41%
	Conjunctival swelling	1	3.70%
	Submandibular lymph node enlargement	1	3.70%
	Swelling of cranial hard palate	1	3.70%
Tumor stage			
	T1	12	44.44%
	T2	4	14.81%
	T3	9	33.33%
	T4	1	3.70%
	NA	1	3.70%

FeLV = feline leukemia virus; FIV = feline immunodeficiency virus; DSH = domestic shorthair; NA, not available.

**Table 2:** All patients with CD3 and CD20 expression and immunophenotype finding.

No:	Immunophenotype	% Area CD3	% Area CD20	Score CD3	Score CD20
1	B-cell	1.50%	26.44%	(−)	(+)
2	B-cell	12.92%	27.97%	(−)	(+)
3	B-cell	15.74%	28.44%	(−)	(+)
4	B-cell	8.93%	29.40%	(−)	(+)
5	B-cell	13.27%	40.15%	(−)	(+)
6	B-cell	3.26%	50.85%	(−)	(++)
7	B-cell	19.25%	53.68%	(−)	(++)
8	B-cell	4.76%	54.66%	(−)	(++)
9	B-cell	6.59%	57.13%	(−)	(++)
10	B-cell	18.74%	58.06%	(−)	(++)
11	B-cell	4.45%	63.54%	(−)	(++)
12	B cell	17.72%	64.95%	(−)	(++)
13	B-cell	2.43%	68.75%	(−)	(++)
14	B-cell	3.00%	70.43%	(−)	(++)
15	B-cell	1.43%	70.72%	(−)	(++)
16	B-cell	0.46%	71.39%	(−)	(++)
17	B-cell	2.02%	71.42%	(−)	(++)
18	B-cell	11.83%	73.42%	(−)	(++)
19	B-cell	2.26%	75.68%	(−)	(+++)
20	B-cell	19.82%	78.39%	(−)	(+++)
21	B-cell	0.74%	79.47%	(−)	(+++)
22	B-cell	1.90%	80.61%	(−)	(+++)
23	B-cell	3.70%	83.44%	(−)	(+++)
24	B-cell	1.76%	83.71%	(−)	(+++)
25	B-cell	0.83%	87.31%	(−)	(+++)
26	B-cell	8.35%	89.02%	(−)	(+++)
27	B-cell	14.66%	90.74%	(−)	(++++)

(−), <25% positive; (+), between 25 and <50% positive; (++), between 50 and <75% positive; (+++), between 75 and ≤90% positive; (++++), >90% positive.

**Table 3:** Multivariate analyses of overall survival in treated cats with nasal lymphoma.

Characteristic	Risk Factor	Number	Median Survival (Days)	Multivariate Analyses		
				HR	95% CI	*p*
Stage						
	T1	6	143	0.04	0.00–0.91	0.04
	>T1	7	120			
Treatment						
	COP chemotherapy	9	121	5.00	0.475–52.59	0.18
	Radiotherapy	4	145			
Anemia						
	PCV > 25	8	145	1.04	0.23–4.72	0.96
	PCV ≤ 25	5	120			
CD20 Expression						
	Low Positive	6	91	120.93	3.2–4568.43	0.01
	High Positive	7	214			

CI, confidence interval; HR, hazard ratio.

## Data Availability

The data presented in this study are available within the article. Raw data supporting this study are available from the corresponding author.

## Supplementary Materials

The following supporting information can be downloaded at: https://www.mdpi.com/article/10.3390/ani14071043/s1, Table S1: Hematological and blood chemistry profiles of cats with nasal lymphoma.
