# Supplementary material for: The Expression Levels of CD20 as a Prognostic Value in Feline B-Cell Nasal Lymphoma: A Pilot Study
Source: Animals (Basel). 2024 Mar 29;14(7):1043. doi: 10.3390/ani14071043 (PMC11010812; doi:10.3390/ani14071043)
Supplement: Supplementary file 1 [file animals-14-01043-s001.zip › animals-2883542-supplementary.pdf]

Table S1. Hematological and blood chemistry profiles of cats with nasal lymphoma.

Parameter	The medians of hematological and blood chemistry profiles			p	Reference range
	Before treatment	The second week of treatment	The sixth week of treatment		
PCV (%)	30.1 (24.80-44.10)	26.8 (12.30-30.80)[a]	25.50 (17.60-33.80)[a]	<0.05	30-45
WBC (x1,000/μL)	12.36 (2.29-18.21)	9.8 (2.69-22.07)	7.59 (2.1-35.63)	0.41	5.50-19.50
Creat (mg%)	1.34 (1.02-2.17)	1.47 (0.77-2.60)	1.23 (0.72-2.66)	0.28	0.90-2.20
ALT (IU/L)	32 (17-512)	40 (13-192)	31 (16-90)	0.66	25-97

Results was expressed as medians (range). PCV, packed cell volume; ALT, alanine aminotransferase; Creat, creatinine; WBC, white blood cell count.
